# Displaying epistemic stance through same-turn self-repair in Chinese civil courtroom interaction

**DOI:** 10.3389/fpsyg.2023.1285759

**Published:** 2023-12-20

**Authors:** Jun Xu, Lei Ge

**Affiliations:** English Department, School of Foreign Languages, Hunan University, Changsha, Hunan, China

**Keywords:** same-turn self-repair, repair operations, epistemic stance, conversation analysis, courtroom interaction

## Abstract

Repair, or conversational repair, frequently appears in court proceedings as a vital mechanism sustaining effective communication. Our study presents a conversational analysis of the choices of different operations in the same-turn self-repair and shows how judges, plaintiffs, defendants, and their lawyers deploy those operations based on a model of epistemic stance. The data were drawn from the top five most-viewed videos of Changsha civil courtrooms from March to May, 2019, totaling more than 50,000 words. In the courtroom interaction, replacing and inserting are the most frequently used operations for all participants. In the courtroom cross-examinations, interlocutors use discrepant same-turn self-repair operations to achieve single or multiple communicative goals, such as improving precision, increasing credibility, highlighting their points, skirting questions, and confirming information. Additionally, when the epistemic stance of the trouble source is [K+], speakers employ most same-turn self-repair operations to keep their [K+] epistemic stance by improving precision and increasing credibility of their utterances or use reformatting or inserting to downgrade epistemic stance to [K−] by decreasing the certainty of their utterances. These findings shed light on the understanding of same-turn self-repairs in the institutional interaction, particularly in Chinese civil courtroom interaction.

## 1 Introduction

The civil courtroom interaction mirrors the features of distinct identities in the court proceedings. The Chinese civil courtroom usually consists of six regular stages: pretrial preparation, court opening, court investigation, court debate, court mediation, and court adjudication (Ge and Wang, 2019). In China, the public has access to civil trials,^1^ both live streams and recordings, since 2016. Such service also provides abundant data for us to explore the forms and functions of the same-turn self-repair operations in the Chinese civil courtrooms.

Previous studies on the courtroom discourse have mostly focused on the interactions of judges (e.g., Balcha, 2014). For instance, Heffer (2008) reveals that judges in the United States display distinct types of discourses such as jury instruction and judgment for different legal purposes. However, drawing on the data from Mandarin Chinese civil trials, Han (2011) analyzes a range of Chinese judges in the civil judgments during a judicial reform, while Ge and Wang (2019) examine the discourses of judges in Chinese civil trials through the critical genre analysis and show that the Chinese traditional values of “harmonious society” and “modesty” are combined with Anglo-Saxon judiciary concepts like “human rights” and “the rule of law” in the current trial system. The previous research reveals that the correlation between the self-repair operations and epistemic stance is not sufficiently investigated. For this reason, this article attempts to further explore the same-turn self-repair operations in Chinese civil courtroom discourse in terms of the epistemics model.

In this article, stance is the attitude, judgments, or commitment of speakers toward propositions (e.g., Wang et al., 2022), and “epistemic stance refers to knowledge or belief vis-à-vis some focus of concern, including degrees of Certainty or knowledge, degrees of commitment to truth of propositions, and sources of knowledge, among other epistemic qualities” (Ochs, 1996, p. 410). Also, according to Heritage (2012b), the epistemic stance of [K−] means that a less knowledgeable interlocutor who lacks a piece of information while [K+] suggests that a more knowledgeable co-participant who has that information.

Overall, this article conducts a moment-by-moment sequential analysis of Chinese civil courtroom interaction with the aim of exploring the correlation between the choice of same-turn self-repair operations and the convergence and divergence of the epistemic stance among Chinese judges, plaintiffs, defendants, and their lawyers.

The rest of the article is divided into six parts. Part II presents the literature review on repair and epistemics. Part III depicts the data and research methods of this article. The results are explained in detail in Part IV. To conclude, Part V discusses the implications of this article’s findings, the limitations, and the future research orientations.

## 2 Literature review

Research on the same-turn self-repairs spans a considerable variety of institutional contexts and languages. A comprehensive overview of same-turn self-repair operations (Schegloff, 2013), repair sequences in the courtroom, and epistemics is presented below.

### 2.1 Same-turn self-repair

Repair, the process individuals use to detect and resolve problems of speaking, hearing, and understanding (Albert and de Ruiter, 2018), is ubiquitous in talk-in-interaction. For instance, Chen and Ye (2022) display how L2 learners orient to public repair sequences by drawing on 10 h of videotaped data from an interactive English course through a conversation analytic study. The same-turn self-repair is the most common repair type (Németh, 2012). In same-turn self-repair, the repairable and repairing sequences occur in the same turn, and the repair is performed by the initiator of the repairable (Rieger, 2003). According to Fox et al. (2009), same-turn self-repair is defined as the process by which speakers stop an utterance in progress and then abort, recast, or redo that utterance. Extract (1) shows an example from Hellermann (2009) of a same-turn self-repair.

Extract (1) from Hellermann (2009, Excerpt 5, p. 116).

01 I: a:nd Julian, my first son,

02 J: ah.

03 **→** I: and **I am** uh **he: is** eight.

In this extract, the speaker self-initiates a repair in line 03 (see the arrow; trouble source and same-turn self-repair indicated in bold) and repairs within the same-turn during which the trouble source occurred. In other words, the speaker first produces “I am” when referring to her son. Subsequently, she utters “he is” as a replacement in the same turn, correcting her error in speaking.

One of the central findings of earlier works on repairs concerns the predominance of self-repairs in talk-in-interaction. Schegloff et al. (1977) notice that the occurrence of self-repairs is more common than other-repairs when they analyze the conversational data of the native English speakers. On the other hand, the analysis of interaction of repair sequences suggests that the turns are designed to facilitate self-repairs, or display the speaker’s sensitivity to the appropriateness of self-repairs and the (possible) impropriety of other-repairs (Dingemanse and Enfield, 2015). Additionally, repair tends to occur in the first position in close proximity to the trouble source. Levinson (1983) summarizes a ranking of preference from high to low: self-initiated self-repairs (in the same turn), self-initiated self-repairs (in the transitional space between turns), other-initiated self-repairs, self-initiated other-repairs, and other-initiated other-repairs. In actuality, Schegloff (1992) argues that trouble sources that are not addressed close to their occurrences can lead to serious problems in an exchange. McHoul (1990) claims that the general preference for repair organization, marked by the predominance of self-repairs over other-repairs, does not operate in the classroom discourse.

In terms of the typology of repair operations, by looking into Japanese other-initiated repairs, Hayashi and Hayano (2013) conclude that replacement, deletion, and repetition are the normal conversation repair strategies through conversation analysis. In another conversational analytic study, Jordanian speakers deploy ten self-initiated repair structures: expansion, hesitation, replacement, repetition, abort and restart, abort and abandon, insertion, deletion, meta-repair, and modify order (Mohammad and Al-Harahshe, 2015). Additionally, drawing on the data of English conversations, Schegloff (2013) identifies ten operations: replacing, inserting, deleting, searching, parenthesizing, aborting, sequence jumping, reformatting, and reordering, based on the English data, which has also been found in Chinese corpus (Ma and Gao, 2018). These studies show that replacement, deletion, and repetition are the three most important repair operations across languages, whereas there are different terms for the same repair strategy, such as “modifying order” and “reordering” in different studies. This study also considers the ten operations proposed by Schegloff (2013) in self-initiated self-repairs as the basic framework, since the ten operations in his categorization can be conducive to identifying the same-turn self-repairs in the Chinese civil court interactions. In this study, six operation types have been found to be commonly used in the collected data: replacing, inserting, searching, recycling, deleting, and reformatting.

### 2.2 Repair sequences in courtroom interaction

Particular characteristics of the courtroom interaction as opposed to everyday conversation and other kinds of institutional and professional talk have been studied and documented since the late 1970s (e.g., Pomerantz, 1978). Previous research reveals that questions and answers are pervasive in the courtroom examination (e.g., Heritage and Clayman, 2010). However, more recently, Matoesian (2018) investigates an affective stance in objections through a multimodal analysis of the trial interactions.

For example, Drew (1991) observes the strategies by witnesses in cross-examinations, finding that witnesses rarely use self-repairs because they could decrease the credibility and accuracy of the testimony. Matoesian (1993) discusses the sequence of conversation repairs, claiming that the role of questioners and answerers reverse between an attorney and a witness when the witness initiates a repair. Romaniuk and Ehrlich (2013) note that the same-turn self-repair from the courtroom interaction in which the repair sequence allows participants to address some of the interactional contingencies related to the norms of the courtroom. In other words, the same-turn self-repairs that we focus on “do not seem to be necessary in terms of correcting something that was problematic or mistaken” (Sidnell, 2010, p. 117), but rather serve other interactional purposes. Understanding the management of same-turn self-repairs in the courtroom interaction, therefore, is fundamental in the courtroom research with implications for co-participants to select the proper type of self-repair to achieve convergence or divergence of their epistemic stance in mundane talk-in-interaction.

However, how epistemic stance influences the choice of repair operations is still under-researched, and particularly in Mandarin Chinese courtroom interaction. In this article, an investigation of the function of epistemic stance in courtroom repair operations may shed light on the mechanism of participants’ choices of repair operations.

### 2.3 Epistemics in courtroom interaction

Epistemics has been studied in many contexts (e.g., Grzech, 2021). For example, Stivers et al. (2011) show that the deployment of and reliance on epistemic resources are organized in a way that impacts on social relations through moment-by-moment conversation analysis. Marin-Arrese (2015) compares epistemic stance strategies in journalistic discourse in English and Spanish and finds some similarities and differences in the use of epistemic expressions in both languages. Recently, Bristol and Rossano (2020) reveal that there is gradient texture to ‘epistemic territory’ and that knowledge domains contribute to the acceptability of disagreement in social interactions. Additionally, Heller (2021) investigates epistemic stances in the display of “doing thinking” in children’s collaborative reasoning through a multimodal discourse analysis. Drawing on the data of parliamentary debates in Catalan, Cuenca (2023) looks into the correlation among disagreement, epistemic stance and contrasts through a qualitative analysis. Overall, epistemics plays a critical role in talk-in-interactions.

On top of that, epistemics has also captivated some attention in the study of the courtroom interaction, though not necessarily under that particular label (e.g., Dong, 2013). Despite grammatical changes, previous studies mainly focus on the epistemic expressions in courtrooms, especially witnesses’ testimonies (e.g., powerless language features were perceived as less credible, less truthful, and less trustworthy than speakers who did not use such features). Table 1 shows some studies on epistemic stance in the legal settings.

**Table 1:** Studies on epistemics in courtroom interactions.

Authors	Context	Data analysis	Results
Gibbons (2008)	Common Law courts in Hong Kong	Conversation analysis	The answerer is pressured to answer in the way the questioner wishes by means of a wide range of linguistic resources related to epistemic stance.
Dong (2013)	Trial Techniques Thomas A. Maucet CITI Publishing House No.6 Edition	Systemic Functional Linguistics	The study shows the techniques for the lawyer and the witness to examine or reply in terms of epistemic stance.
Szczyrbak (2021)	A British libel trial	Discourse analysis	Epistemic lexical verbs are used to communicate moderate certainty rather than uncertainty and doubt.

For instance, Szczyrbak (2021) focuses on epistemic lexical verbs, especially “I think,” on the basis of the data from an actual court case in the United Kingdom. It is nevertheless striking to note that she finds that epistemic lexical verbs are used to communicate moderate certainty rather than uncertainty and doubt, and on that basis argues, with reference to Simon-Vandenbergen and Aijmer (2007), that the expressions are deployed to perform various rhetorical functions including persuasion, manipulation, challenging, confrontation, and acceptance. Additionally, in the studies on epistemics in the context of the courtroom interaction, particularly in court proceedings and court debate, interlocutors interact and understand one another on the basis of two aspects: the judgment of epistemic stance of their own and of their interlocutors and the adjustment of action and understanding (Heritage, 2012b). In the courtroom discourse, epistemics stance is a necessary resource for speakers to initiate repairs (Robinson, 2013). Speakers claim their [K+] or [K−] stance in their expertise or real-world experience based on the judgment of their epistemic stance. As an illustration, Table 2 shows the use of distinct types of questions (Sadock, 2012) and the display of epistemic stance.

**Table 2:** The use of distinct types of questions and the display of epistemic stance.

Types of questions	epistemic stance	Examples
Wh-Questions	Unknowing [K−]	What do you think this is?
Polar and alternative questions	Unknowing [K−]	Is poo-poo one word, or two?
Tag questions	Unknowing [K−]	You are not going to start any fires, are you?
Rhetorical Questions	Knowing [K+]	Is the government very successful? No.

This article aims to show how participants express their epistemic stance by employing different same-turn self-repair operations based on their understandings of their own epistemic stance and of their interlocutors by looking into the use of repair operations in the Chinese civil courtroom. As such, we address the following specific questions.

1. What are the most commonly used forms and functions of same-turn self-repair operations in Chinese civil courtroom interaction?
2. How does the same-turn self-repair operate in terms of the model of epistemic stance within the conversation analysis framework?

## 3 Data and research methods

We conducted our data analysis in four successive steps: transcription, coding, quantitative analyses of the entire data set, and turn-by-turn analysis of the repair sequences in the light of epistemics.

### 3.1 Data collection

The data in this article were drawn on the natural interaction in the typical civil cases from the intermediate court of Changsha, including two contract dispute cases, two labor dispute cases and one loan dispute case, attracting the top five hits from March to May, 2019, with a total of more than 50,000 words. Five cases were selected to examine repair sequences in the civil courtroom interaction. The reason that the top 5 most-viewed cases were chosen is that they might be representative of the local civil court interaction, and participants in the civil courtroom can also be typical of the local people in the data chosen. Case 1 was a commercial apartment pre-sale contract dispute case, in which the real estate company was accused of cheating customers in the contract. Case 2 was a labor contract dispute case, in which the defendant was accused of requesting unreasonable compensation from the previous employed company. Case 3 was a commercial apartment pre-sale contract dispute case, in which the real estate company was accused of delaying the completion date and violating the regulation of renovation in the contract by the four homeowners. Case 4 was a labor contract dispute case, in which the company is accused of dismissing employee without compensation. Case 5 was a loan dispute case, in which the plaintiff asked for the money back. All personal names in the corpus and other references that could allow the disclosure of sensitive information have been replaced with pseudonyms. Table 2 shows the distribution of different repair operations deployed by all participants in the civil trials.

Overall, 324 same-turn self-repairs were produced by judges, plaintiffs, defendants, and their lawyers in the civil courtroom interaction. Table 2 shows that replacing and inserting are the most frequently used operations in the same-turn self-repair, altogether accounting for more than 50%. Searching and recycling make up 19.6 and 18.63% respectively, followed by deleting (8.50%) and reformatting (2.94%).

Also, Table 3 shows the frequency of the six different same-turn self-repair deployed by judges, plaintiffs, and defendants in the data.

**Table 3:** Frequency of same-turn self-repair operations.

Participants	Replacing	Inserting	Searching	Recycling	Deleting	Reformatting
Judges	23.08%	30.77%	15.38%	13.46%	5.77%	11.54%
Plaintiffs	25%	23.53%	19.85%	22.79%	8.09%	0.74%
Defendants	27.59%	22.41%	21.55%	16.38%	10.34%	1.72%
Total	75.67%	76.71%	56.78%	52.63%	24.2%	14%

Different from Tables 3, 4 compares the frequency of six self-repair operations deployed by the major participants, including judges, plaintiffs, and defendants, in the data, with a total of 203 (excluding 121 self-repairs produced by the lawyers of plaintiffs and defendants). Similarly, it is manifest that replacing and inserting are the most frequently used operations for all participants in courtroom interactions. The only difference is that inserting is the most used operation by judges (30.77%), while plaintiffs (25%) and defendants (27.59%) choose replacing as the most frequently used repair operation in same-turn self-repairs. Additionally, recycling and searching are employed more by defendants and plaintiffs than by judges. A final striking point is that there is a difference among participants in the least frequently used operations. Specifically, reformatting is the least deployed by plaintiffs (0.74%) and defendants (1.72%), while deletion is the least used by judges (5.77%). One possible reason that reformatting is least common among plaintiffs and defendants is that they are average citizens with accents when they speak Mandarin Chinese. In other words, they are not well-educated in contrast to judges so that they do not alter the syntactic constructions to upgrade or downgrade their epistemic stance. Another reason is related to the property of the Chinese language, which is considered as a paratactic language (Li and Yu, 2021), a language in which logical and semantic relationships between elements within sentences are usually implied and understood from context rather than expressed lexically.

**Table 4:** Distribution of repair operations in Chinese civil courtroom.

Repair operations	Numbers	Percentages
Replacing	83	25.49%
Inserting	81	24.84%
Searching	64	19.6%
Recycling	61	18.63%
Deleting	28	8.5%
Reformatting	7	2.94%
Total	324	100%

### 3.2 Data analysis

At the outset, all instances of same-turn self-repair configuration types were collected for detailed analyses. By and large, 324 same-turn self-repairs were produced by the judges, plaintiffs, defendants, and their lawyers in the five cases.

Secondly, those selected instances were transcribed in greater details. In addition to talk, inhales, exhales, pauses, and sound stretches were transcribed as well for interactional meanings and are not extraneous elements of talk-in-interaction (Wong and Waring, 2010). In conversations, verbal and nonverbal means (e.g., eye gaze and gestures) of communication are deployed conjointly to convey speakers’ intentions.

Finally, all examples were grouped into different types of repair operations in light of the categorization given by Schegloff (2013). The results are rechecked, and the time interval between the categorizations is one week and the results are nearly the same. It is found that there are six most common types of same-turn self-repair operations deployed in the Chinese civil courtroom interaction, namely replacing, inserting, searching, recycling, deleting, and reformatting.

There are several benefits in investigating repair sequences from a CA perspective. Firstly, researchers from the CA perspective regard repair sequences as a means to achieve mutual understanding. Their focus of investigation is naturally occurring interactions that involve the negotiation of meaning. In other words, the CA perspective offers more of a holistic understanding of communication by examining sequentially organized actions. CA investigation into courtroom self-repairs considers the same turn organization of interaction moment-by-moment, thus providing a more progressive view of same-turn self-repairs.

Secondly, a CA approach to the study of repair sequences can be fruitful to the courtroom research because it explores naturally occurring conversations and can, therefore, shed light on participants’ spontaneous ways to correct, clarify, or confirm each other’s utterances. In the courtroom interaction, language is a vehicle for the interaction but not its focus. Researchers from a CA perspective contend that courtrooms create an environment conducive to exploring repair sequences because these operations provide speakers with ample opportunities to modify their utterances (Mininni et al., 2014). The CA approach to repair sequences investigates how repair is relevant to the participants by identifying the object of repair, or trouble-source (Wong and Waring, 2010, p. 213), and by investigating repairs in terms of repair initiations and repair operations. This article attempts to advance our understanding of a relatively unexamined domain: the various types of self-initiated same-turn self-repair operations that participants employ as they engage in a conversation in the Chinese civil courtroom from a conversation analytic perspective.

Finally, there are three major steps in using conversation analysis in the study of repair sequences. One fundamental step is that 324 same-turn self-repairs sequences were collected, followed by an examination of variation among coders. In terms of inter-rater reliability of two independent coders, only a small number of 324 same-turn self-repairs are identified in five civil cases. There is little incongruency between raters, and the percentage agreement is almost 100%. If there is a minor incongruency, both raters would discuss it until they reached agreements. The last step is to provide a moment-by-moment analysis of the repair sequence to address the research questions. It should be noted that both authors have received intensive training in coding and transcribing the data in this study, and they have also published monographs and journal papers within the conversation analytic framework.

However, CA research does not generally report precise numbers, but instead relies on informal quantification (Schegloff, 1993) or numeric descriptors (Stivers, 2015). CA investigations of self-repair operation types have reported their data in terms of their relative frequencies in their data set, such as such as over-represented, extremely common (Fox et al., 2010), significantly more (Bada, 2010), a higher proportion (Quan and Weisser, 2015, p. 46), a considerable number (Németh, 2012, p. 2,032) and far less common (Schegloff, 2013, p. 47). Also, Schegloff (1993) argues that single case analysis can demonstrate the orderly operations in conversation and talk-in-interaction and quantification can be seen as the multiples or aggregates of single cases. In other words, the qualitative analysis of single episodes of talk in interaction can display the way in which the self-repair operation unfolds turn by turn and the sequential environment in which self-repairs occur within the conversational analytic framework.

## 4 Results

The data clearly show that judges, plaintiffs, defendants, and their lawyers have deployed same-turn self-repair operations in the Chinese civil courtroom to display epistemic stance. Also, speakers can choose different operations to express their epistemic stance and achieve a specific interactive goal in various contexts. More importantly, epistemic stance is dynamic in the same-turn self-repairs, which can be roughly divided into two categories: the convergence and divergence in the correlation between the epistemic stance in trouble source and the epistemic stance after employing same-turn self-repair operations. Consequently, a model of epistemic stance in same-turn self-repairs is constructed with an aim of illustrating how speakers choose same-turn self-repair operations to achieve the interactive goals from the perspective of epistemics.

In the Chinese civil courtroom, judges perform the basic role, namely, the organizer of courtrooms, who has [K+] epistemic stance in the professional knowledge of court regulation and law enforcement. Plaintiffs and defendants enjoy [K+] epistemic stance when they recount their narratives in relation to the civil case. Plaintiffs and defendants present their versions of the dispute with evidence to convince the judge that their version of events is the most credible and accurate. Therefore, judges deploy same-turn self-repair operations less frequently than plaintiffs, defendants, and their lawyers, and the choice of plaintiffs’ and defendants’ same-turn self-repairs operations has many similarities.

**Figure 1:**
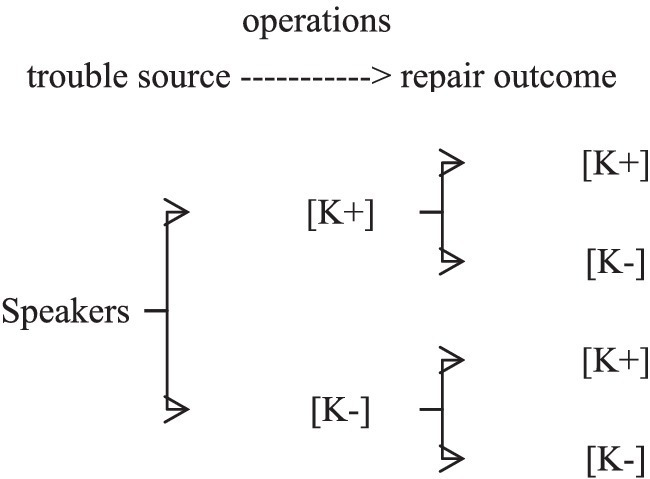
A model of epistemic stance in same-turn self-repairs.

Besides, the speaker’s epistemic stance in the same-turn self-repair is dynamic. Although speakers deploy different same-turn same-repair operations to express [K+] or [K−] epistemic stance, there are four models of speakers’ epistemic stance to achieve single or multiple interactional purposes in the Chinese civil courtroom interaction by employing different same-turn self-repair operations, as shown in Figure 1.

As Figure 1 shows, the first category of changing epistemic stance is the convergence of epistemic stance. Usually, participants in the civil courtroom deploy most same-turn self-repair operations to keep the convergence of their [K+] epistemic stance by improving the accuracy of utterances, highlighting the points of their claims. However, speakers recycle full or partial questions to maintain the convergence [K−] epistemic stance, urging recipients to give responses.

Conversely, the divergence of speakers’ epistemic stance is the other category. Speakers sometimes employ repair operations to express [K−] epistemic stance to decrease the certainty of claims by inserting, deleting, and reformatting, when their trouble sources reflect speakers’ [K+] epistemic stance. When speakers’ trouble sources display [K−] epistemic stance, judges have more opportunities to abort the initial question and restart with a new syntactic form upgrading epistemic stance to [K+].

In the following sections, the two categories including four ways of changing epistemic stance will be illustrated one by one with contextualized examples of the three participants’ same-turn self-repair based on a series of selected excerpts.

### 4.1 The convergence of epistemic stance in same-turn self-repair

The convergence of epistemic stance in same-turn self-repairs in the Chinese civil courtroom can be expounded in two ways. Firstly, in Chinese civil courtroom, predominant same-turn self-repair operations are deployed by all participants to keep the convergence of [K+] epistemic stance. As an illustration, judges prefer inserting, replacing, and searching to provide clear instructions and professional knowledge, displaying [K+] epistemic stance, thus positioning in the [K+] epistemic stance in the courtroom context. While defendants, plaintiffs, and their lawyers claim the [K+] epistemic stance in the trial narrative versions of reality, they would express their [K+] epistemic stance to eliminate troubles and increase the degrees of credibility and accuracy of their statements about the competing versions of narratives by adopting repair operations. Secondly, the convergence of [K−] epistemic stance is mainly found in judges’ same-turn self-repairs, employing recycling asserting [K−] epistemic stance. Generally, a request for information positions the requester with an unknowing [K−] epistemic stance and the recipient with a knowing [K+] epistemic stance. Interactants express their [K−] epistemic stance to increase the pressure for the recipient to respond promptly by recycling.

#### 4.1.1 The convergence of [K+] epistemic stance

**Figure 2:**
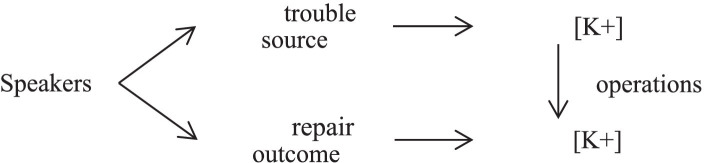
The convergence of [K+] epistemic stance.

The predominant same-turn self-repairs in Chinese civil courtroom interaction aim to keep the convergence of [K+] epistemic stance. The convergence of [K+] epistemic stance means that speakers express [K+] epistemic stance before and after employing same-turn self-repair operations, which is clearly illustrated by Figure 2.

Speakers, who enjoy [K+] epistemic stance, tend to alter their turns to facilitate their listeners’ understanding by expressing their [K+] epistemic stance. The main function of most same-turn self-repair operations is to increase the accuracy and credibility by implementing a [K+] action to claim the interlocutor’s [K+] epistemic stance. For example, the lawyer of the plaintiff who enjoys the [K+] epistemic stance in the trial narrative prefers asserting [K+] epistemic stance to offer an accurate narrative by means of error replacing.

**Extract (2) Error Replacing (** http://tingshen.court.gov.cn/live/5308919 **).**

01 Plaintiff’s lawyer: X 的 收 房 时间 也 是 13年12月15 号 左右.

de shou fang shijian ye shi nian yue hao zuoyou.

MM own home time also COP year month day or so.

The date when Mr. Fan owned the home was also roughly 15 December, 2013.

(noises)

02 Judge: 不 要 插话。 房间 里 的 人, 不 要 讲。.

bu. yao chahuo fangjian li de ren bu. yao jiang.

NEG MV interrupt room Prep. MM person NEG MV talk.

“No interruption. People in the courtroom are not allowed to talk.”

03 Plaintiff’s lawyer: 啊, 对, 左右 啊, 左右.

a dui zuoyou a zuoyou.

ah correct roughly ah roughly.

“Ah, it is correct. Roughly, ah, roughly.

04 具体 应该 由 被告, 应当 由 被告 提交 原告.

juti yinggai you beigao yingdang you beigao tijiao yuangao.

specifically MV by defendant MV by defendant submit plaintiff.

Specifically, the defendant should, the defendant should submit.

05 签字 的, 房屋 交接, 这 个 表, 予以 核实 啊.

qianzi de fangwu jiaojie zhe ge biao yuyi hehsi a.

sign MM house handover this CLF form give confirm SFP.

the house handover form signed by the plaintiff. This should be confirmed,

06 因为 这个 时间 跨度 长,.

yinwei zhege shijian kuadu chang.

because this time span long.

because this involves an extended period of time.

07 **→** 应当 是 **由**, 不 是 **有**, 提交 房屋 交接 表.

yingdang shi you bu. you you tijiao fangwu jiaojie biao.

MV COP Prep. NEG COP have submit house handover form.

The word should be “you” (with a rising tone), not “you” (with a falling-rising tone), to submit the handover form.

In Extract (2), the judge tends to confirm the date of X’s ownership of the house with the plaintiff’s lawyer. Prior to the segment, both the plaintiff and the defendant mention the house’s completion date and the home ownership form. Thus, the judge requests two parities to submit their originals and checks the date. The plaintiff’s lawyer responds in line 01 and adds details to prove his claims (line 04). In this extract, the phonetic errors are made by the lawyer, who replaces the mispronounced word with the corrected one. Interlocutors repair phonetic errors to ensure their accuracy and credibility of the statements, which is a [K+] self-repair action.

Usually, phonetic errors are slips of tongues. The phonetic errors in the Chinese civil courtroom appear not only in the syllables, but also in tone. In the case of *you* (by) in line 07, the plaintiff’s lawyer intends to supply details to prove the statement in line 04 and enhance his credibility. To be specific, he mispronounces *you* with a falling-rising tone (“*you*”) and replaces with a rising tone (“*you*”). A sound stretch initiates the repair when the plaintiff’s lawyer realizes his tone error (a falling-rising tone), which is followed by the correct tone of “*you”* (a rising tone).

The plaintiff’s lawyer claims the [K+] epistemic stance in the trial narrative, and he would express his [K+] epistemic stance to eliminate troubles and increase the degree of credibility and accuracy of his statements by replacing errors. The following example will demonstrate how participants employ various subtype same-turn self-repair operations, such as inserting (extract 3) to maintain the convergence of [K+] epistemic stance, hence achieving interactional goals.

**Extract (3) Inserting (** http://tingshen.court.gov.cn/live/5308919 **).**

01 Judge: 讲 慢点, 这里, 真实性 关联性 无 异议.

jiang mandia zheli zhenshixing guanlianxing wu yiyi.

speak slowly here authenticity relevance NEG disagreement.

Speak more slowly, here. (You) agree with authenticity and relevance.

02 只 对 合法性 有 异议 是 吧?

zhi dui hefaxing you yiyi shi ba.

only Prep. legality have disagreement COP QP.

You only doubt the legality (of the evidence), do not you?

03 Defendant’s lawyer: 恩. 没有 原告 本人 签字 授权.

en meiyou yuangao benren qianzi shouquan.

eh NEG plaintiff self sign authorize.

“Eh. The letter was not signed and authorized by the plaintiff.

(clearing voices) (5 s)

04 **→** 并且 函件 内容 对 原告 **合同 条款**::

bingqie hanjian neirong dui yuangao *hetong tiaokuan::*

and letter content Prep. plaintiff contract terms.

and in terms of the content of the letter, the terms in the contract,

05 **→** 合同 **关键** 条款 陈述 有 错误.

*hetong guanjian tiaokuan* chengshu you cuowu.

contract critical terms statement have mistakes.

the statement of the **critical** terms in the contract signed by the plaintiff was mistaken.”

In Extract (3), the judge organizes the cross-examination about the fourth piece of evidence (lines 01 and 05). Retrospectively, the plaintiff intends to prove the fact that he has required the compensation for the house for an extended period. However, the defendant’ lawyer disagrees with the legality of the evidence, claiming that the letter does not have the authorized signature of the plaintiff and has inaccurate statement of contract terms (lines 03–05). The defendant’s lawyer stresses that the inaccurate statement of the contact is serious and proves the illegality of the evidence by inserting *guanjian* (key) before *tiaokuan* (term) in line 05. In this instance, the speaker asserts his [K+] epistemic stance in the trial narrative by employing inserting self-repair operation in the same-turn. The same-turn self-repair is initiated with a cut-off or a sound stretch, and then the adjective (critical) is inserted in line 05, which aims to accentuate the importance of the point in question.

#### 4.1.2 The convergence of [K−] epistemic stance

**Figure 3:**
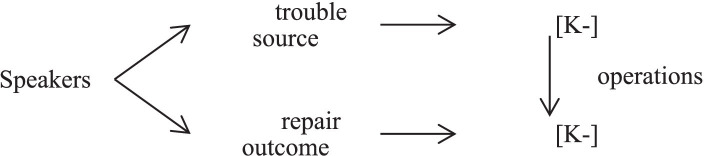
The convergence of [K−] epistemic stance.

The convergence of [K−] epistemic stance means that speakers express [K−] epistemic stance before and after employing same-turn self-repair operation, which can be clearly understood by looking at Figure 3.

A request for information positions the requester as asserting an unknowing [K−] epistemic stance and the recipient as claiming a knowing [K+] epistemic stance. In fact, the judge employs same-turn self-repair operations to keep the convergence [K−] epistemic stance. Through partial or full recycling, speakers assert their [K−] epistemic stance to urge the respondent to give a reply.

Extract (4) below is a typical example of recycling by the plaintiff, who is not well-educated and speaks without a clear logic or accurate grammar. Another striking point is that he delivers his trial narrative in Mandarin Chinese with a strong accent.

**Extract (4)** Recycling (http://tingshen.court.gov.cn/live/4702467).

01 Judge: 16 年 4月 21号 的 这 5万 块钱 还给 你 没有?

nian yue hao de zhe wan kuaiqian huangei ni meiyou.

year month day MM this 10,000 yuan return 2PS NEG.

Did you get the 50,000 yuan back on April 21, 2016?

02 Plaintiff: 还 了。 (6 s).

huan le.

return PRT.

“Yes, he did.”

03 Judge: 那 为什么 借条 原件 还 在 你 手上 呢?

na weishenme jietiao yuanjian hai zai ni shoushang ne?

then why loan receipt original still Prep. 2PS hand QP.

“Then, why did you still have the original loan receipt in your hand?

04 Plaintiff: 这个:: 是 他 当事人 他 认为 这个 钱 他 借给 谁 去 了。.

zhege:: shi ta dangshiren ta renwei zhege qian ta jiegei shui qu le.

this:: COP 3PS party 3PS think this money 3PS lend who go ASP.

This::, the party involved, he doubted who borrowed the money.

05 **→** 他 向 我 来 要 钱, **到底 到底** 是 不 是 借给 他 了,.

ta xiang wo lai yao qian daodi daodi shi bu. shi jiegei ta le.

3PS Prep. 1PS come ask money really really COP NEG COP lend 3PS ASP.

He asked me for money. Did I really lend money to him?

06 他 就 把 这个 条子 拿 出去 了,.

ta jiu ba zhege tiaozi na chuqu le.

3PS just Prep. this receipt take away ASP.

He took this receipt away.

Extract (4) is an instance of lexical recycling, by which the plaintiff displays his [K−] epistemic (Line 05). The judge begins the talk by mentioning the first question to confirm whether the money is paid back or not (line 01). Then, the plaintiff gives a positive response, “Yes, he did,” in line 02. The judge raises the second question why the receipt is still in his hand (in line 03). However, the plaintiff has trouble in understanding and utters irrelevant information in line 04. The word recycling “really” (“*daodi*” in line 05) is treated as implementing a self-repair to keep the convergence [K−] epistemic stance in the details of the case. The plaintiff deploys a recycling to keep the convergence [K−] epistemic stance in the turn to highlight the detail.

### 4.2 The divergence of epistemic stance in same-turn self-repair

The divergence of epistemic stance in same-turn self-repairs in Chinese civil courtroom also includes two ways: [K+] to [K−] or [K−] to [K+]. In terms of the gradients in the formulation of epistemic stance, the fundamental observation is that unknowing speakers ask questions and knowing speakers make assertions (Heritage, 2012a). The gradient from [K+] to [K−] is reflected from syntactical forms as follows: declarative syntax > declarative syntax with a final rising intonation > tag questions > negative interrogative syntax > interrogative syntax. In addition to the gradients, whether utterances are to be understood as requesting or conveying information depends on syntax and intonation, which represents the epistemic stance that speakers assert or attribute to themselves and their recipients at a given point in an interaction.

Given that civil courtroom interaction is organized primarily around question-answer sequences (Heritage and Clayman, 2010), it is perhaps not surprising that same-turn self-repairs are deployed by judges in a way that conforms to constraints on asking questions. While it is generally known that the turn-taking system governing courtroom interaction restricts judges’ turns-at-talk to raise questions, what is perhaps less manifest is the fact that there are also constraints on the form that judges’ questions can take. Indeed, judges use reformatting, deleting, replacing to modify the grammatical format of questions that seemingly satisfy one such constraint in the context. Plaintiffs, defendants, and their lawyers self-repair their utterances to maintain their preferred versions of narratives of reality. However, they would downgrade the epistemic stance from [K+] to [K−] to decrease the definitiveness of claims and responses, obfuscating responsibilities, or skirting questions by employing inserting and deleting to adjust their epistemic stance.

#### 4.2.1 The divergence of epistemic stance from [K+] to [K−]

**Figure 4:**
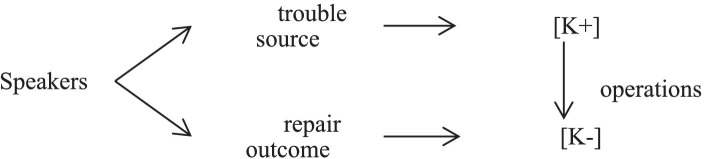
The divergence of epistemic stance from [K+] to [K−].

The divergence of epistemic stance from [K+] to [K−] means that speakers allege [K+] epistemic stance in their original utterances, but assert [K−] epistemic stance after employing same-turn self-repair operations. Figure 4 makes the procedure easier to understand.

Although judges could upgrade epistemic stance in the knowledge of the current trial after they have obtained competing versions of narrative from both parties. Judges prefer confirming or rechecking the information to verify the key issues. By way of illustration, they would repeat or restate the utterance in a declarative form, which positions [K+] epistemic stance, and subsequently delete redundant elements with a rising intonation, downgrading the epistemic stance.

Judges employ reformatting to alter a declarative syntax, which positions [K+] epistemic stance, to an interrogative syntax asserting [K−] epistemic stance. Generally, judges add interrogative words to or insert tag questions into the declarative construction due to the interactional import.

**Extract (5) Inserting (** http://tingshen.court.gov.cn/live/5325594 **).**

01**→**Judge: 我 问 一 下, 这个, 原告 方 第一 个 问题.

wo wen yi xia zhege yuangao fang diyi ge wenti.

1PS ask one CLF this plaintiff party first CLF question.

“I ask the plaintiff the first question.

02 啊,你们 买 房 的 时候, 看 了 他 这个 房屋 销售.

a, nimen mai fang de shihou kan le ta zhege fangwu xiaoshou.

ah 2PP buy home MM time see ASP 3PS this house sale.

Ah, when you bought the house, did you see the plan of this house?

(raising his right hand)

03 这 个 图 没有? 就是 他 所说 的 这 种 图, 表格.

zhe ge tu meiyou jiushi ta suoshuo de zhe zhong tu biaoge.

this GLF plan NEG just 3PS say MM this CLF plan, table.

That is, the plan or the table he mentioned.

04 就 这 种 户型 图 啊? (raising his right hand with the paper).

jiu zhe zhong huxing tu a.

just this CLF floor plan QP.

Just like this sort of floor plan?

05 有 没 有?

you mei you.

have NEG have.

did not you?

06 Plaintiff’s lawyer: 户型 图 应当 是 看 过 [但是::]

huxing tu:: yingdang shi kan guo, [danshi::]

floor plan:: MV COP see ASP [but::]

I should have seen the floor plan, [but::]

07 Judge: [看 过]↓ **是 吧?**

[kan guo]**↓** shi ba**?**

[see ASP]**↓**right QP

(You) [saw] it-**↓did not you?**

08 Plaintiff’s lawyer: 但是**↓** 我 不 知道 看 的 户型 图 是 哪 一 种.

danshi wo bu. zhidao kan de huxing tu shi na yi zhong.

but 1PS NEG know see MM floor plan COP which one CLF.

“However, I am not sure which type of floor plan.

09 肯定 是 没 有 层高 的.

kending shi mei you cenggao de.

definitely COP NEG have floor height MM.

Definitely, (the floor plan) does not show the floor height.

10 肯定 是, 只有 这个,就 说 是 几 室 几 间,.

kending shi zhiyou shege jiu shuo shi ji shi ji jian.

definitely COP only this just say COP several room several room.

Definitely, yes. Only this, that is to say, with several rooms.

11 类似 那种 的。.

leisi nahzong de.

like sort of MM.

sort of.”

12 Judge: 哦。.

o

oh

“Oh.”

13 Plaintiff’s lawyer: 肯定 是 没有 层高 指示.

kending shi meiyou cenggao zhishi.

definitely COP NEG floor height mark.

“Definitely, there was no floor height marked.

14 Judge: 看 过 但是 那种 没有 层高 的 **(0.5) 是 不 是?**

kan guo danshi nazhong meiyou cenggao de *shi bu shi*?

see ASP but that NEG floor height MM COP NEG COP.

You saw the floor plan but without floor height**, (0.5) did not you?**

15 Plaintiff’s lawyer: 对。.

dui.

right.

Yes.

In extract (5), the judge checks whether the plaintiff has seen the floor plan. In this context, the plaintiff’s lawyer is adopting [K+] epistemic stance, and judge positions [K−] epistemic stance. The judge claims an epistemic stance change from [K+] to [K−] in line 01, who is adopting a particular epistemic stance [K+] toward what is being verified. He nevertheless abandons the declarative construction by a cut-off and selects instead what is perhaps a more cautious interrogative [K−] stance, more cautious insofar as the defendant assigns epistemic primacy, which is the [K+] stance to the plaintiff. When getting the answer from defendant in line 07, the judge repeats the verb – *kanguo* (saw), upgrading the epistemic stance toward [K+], and subsequently downgrades the epistemic stance with a tag question toward [K−]. Therefore, the shift of epistemic stance in line 07 is from [K+] to [K−].

In line 13, the plaintiff’s lawyer emphasizes that he did not see the floor-to-ceiling height. The judge repeats the answer in line 14 and after 0.5-s pause initiates a repair with a tag question — *shi bu shi* (did not you) in the final position of the turn. Similarly, a change of epistemic stance from [K+] to [K−] occurs in line 14 concerning the trial narrative. In terms of reformatting, the judge begins his turn with a declarative form. Before completing that declarative, he enjoys a [K+] stance which he substitutes with an interrogative form or a tag question by inserting *shi bu shi* in line 14 and downgrades epistemic stance from [K+] to [K−], ceding the primary epistemic stance to his recipient.

**Figure 5:**
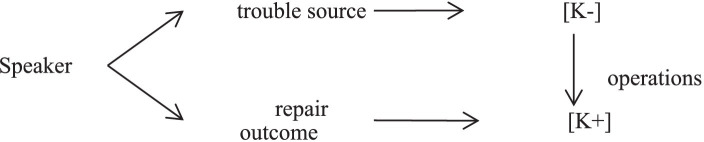
The divergence of epistemic stance from [K−] to [K+].

It is also worth noting that the judge raises his right hand with the paper in line 04 to draw the plaintiff’s lawyer to the floor plan in his hand, which is illustrated in Figure 5. This waving gesture is employed to highlight the topic in question. At the same time, the judge also oriented himself toward the co-participant in the moment-by-moment unfolding interaction.

#### 4.2.2 The divergence of epistemic stance from [K−] to [K+]

**Figure 6:**
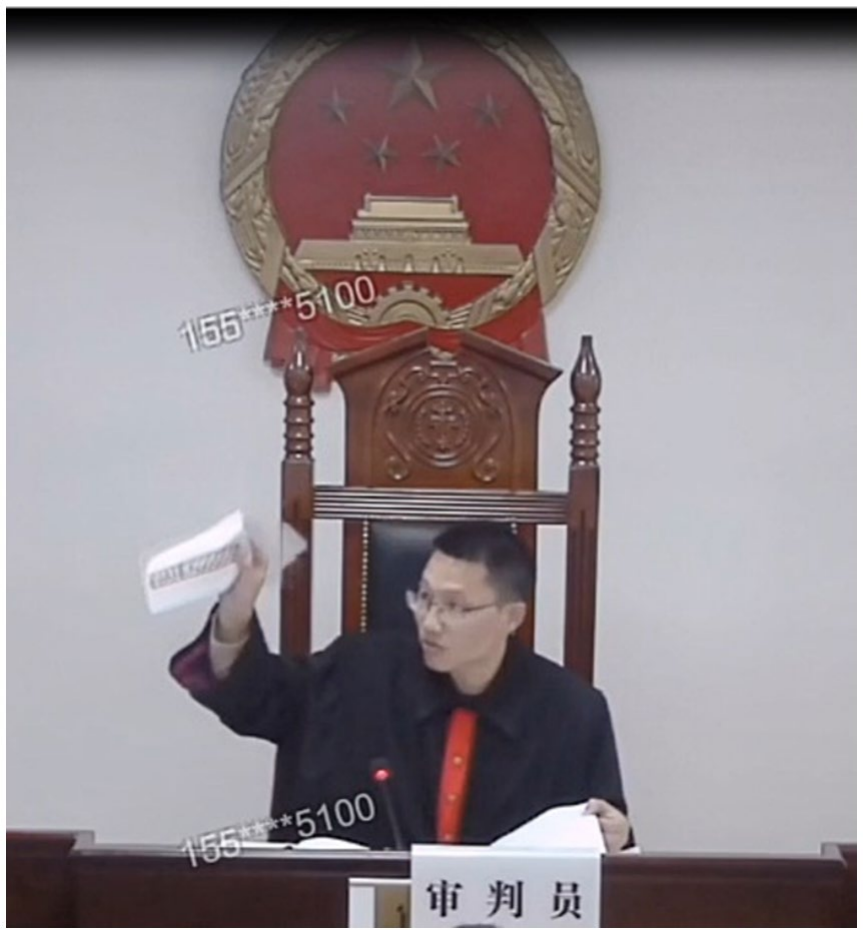
A waving gesture of the judge. Reproduced from http://tingshen.court.gov.cn/live/5325594 with permission from The People’s Court of the Yuelu District, Changsha City, Hunan Province, P. R. China.

The divergence from epistemic stance [K−] to [K+] means that speakers express epistemic stance from [K+] in their original utterances to [K−] after employing same-turn self-repair operations. The detailed process is shown as Figure 6.

Since judges are supposed to enjoy more power in the courtroom, they have more opportunities to change their epistemic stance when they are in [K−] epistemic stance. Generally, judges abort the initially more open-ended and less “leading” questions (i.e., wh-format), and then restart the question with a new syntactic construction, which indicates a particular answer to the recipient (i.e., yes or no). In the collected data, this way the epistemic stance changes mainly appears in judges’ same-turn self-repairs. Judges upgrade their epistemic stance from [K−] to [K+] to narrow down the response scope and push recipients to reply by reformatting (extract 6) to upgrade the constraints on asking for information.

**Extract (6) Reformatting (** http://tingshen.court.gov.cn/live/5325594 **).**

01 Judge: 第三 一 个 问 一 下, 在17年, 他 约定 的 是.

disan yi ge wen yi xia zai nian ta yueding de shi.

third one CLF ask one time Prep. year 3PS promise MM COP.

“Thirdly, I confirm one point. In 2017, his promise was.

02 7月份 交 房 嘛, 交 房 的 时候,

yuefen jian fang ma jiao fang de shihou.

month handover house SFP handover house MM time.

The home handover was due in July. During the handover,

03 你们 当时, 交 房 时, 你们 当时.

nimen dangshi jiao fang shi nimen dangshi.

2PP when handover home time 2PP when.

At that time, during the handover; at that time,

04 **→** 对 层高 提出 异议 没 有?

dui cenggao tichu yiyi mei you?

Prep. floor height raise disagreement NEG have.

did you question the floor height?

05 Plaintiff’s lawyer: 提出 了。.

tichu le.

raise PRT.

“Yes, I did.”

06 **→** Judge: 当时, 就 提出 了,.

dangshi jiu tichu le.

then just point out ASP.

“Then, you pointed it out.”

07 Plaintiff’s lawyer: 对。.

dui

right.

“Right.”

**08 →** Judge: 以 **什么** 形式 啊? 以 **书面** 形式 还是 什么?

yi shenme xingshi a yi *shumian* xingshi haishi shenme.

Prep. what form QP with written form or what.

“Which form? **Written** form or others?”

09 Plaintiff’s lawyer: 额,包括,当面 也 提 了, 书面 形式 也 提 了。.

e baokuo dangmian ye ti le, shumian xingshi ye ti le.

eh include face-to-face also raise PRT written form also raise PRT.

Erm, including, I point it out in person and in the written form.

10 包括 后面, 我们 交 的 那 两 个 报告,那个.

baokuo houmian women jiao de na liang ge baogao nage.

include latter 1PP submit those two CLF report sort of.

including the latter, those two reports we submitted, sort of.”

Extract (6) is drawn from the court examination. Prior to the segment, the court discussion concentrates on the first controversial issue -- the floor-to-ceiling height. The judge wants to know whether the plaintiff checks the floor height when he takes the house (line 01). Moreover, he proposes the second question about the form of the complaint (line 08). Generally, the objection can be raised in two forms: spoken or written. To prevent the possible misunderstanding of the *xingshi* (form), the judge inserts *shumian* (written) before the trouble source. The inserting repair not only inserts an adjective word, but also reformulates a wh-question as a yes-no question, which serves to prevent the possibility of misunderstanding and provide details of the question. The wh-question interrogation shows that the judge positions in the [K−] epistemic stance in the trial narrative. However, he moves from a [K−] stance toward [K+] by increasing the certainty of respondents’ answer.

Summarily, same-turn self-repairs are usually solicited by asserting the convergence of speakers’ epistemic stance of [K+] or [K−] or changing the epistemic stance of interlocutors, which can be embodied by two formats: [K−] to [K+] and [K+] to [K−]. The convergence of [K+] epistemic stance is the principal way in the model of epistemic stance of same-turn self-repairs to achieving the fundamental interactional goal – improving the accuracy of utterances in the trial narrative. Speakers employ most operations to keep the convergence of [K+] epistemic stance, such as replacing (extract 2) and inserting (extract 3). Judges improve the accuracy of their utterances to provide more authenticity and professional instructions, asserting [K+] epistemic stance in the same-turn self-repairs. While plaintiffs, defendants and their lawyers improve the accuracy of their statements to increase the degree of credibility and highlight the key points to accentuate their version of narratives, claiming their [K+] epistemic stance in the same-turn self-repairs. Judges have more opportunities to keep the convergence [K−] epistemic stance in same-turn self-repairs, and they recycle partial or full questions (extract 5) to express his [K−] epistemic stance, imposing the question, and urging respondent to give answer.

While defendants, plaintiffs, and their lawyers enjoy the [K+] epistemic stance in their versions of trial narratives of reality, they may also downgrade epistemic stance [K+] to [K−] to decrease the degree of certainty to avoid judges’ questions or shirk responsibilities by adjusting epistemic stance by inserting (extract 5). Judges choose reformatting (extract 6) to upgrade epistemic stance from [K−] to [K+].

Unlike previous studies, which mostly center on the judge’s discourse (e.g., Han, 2011), this study explores how judges, plaintiffs, defendants, and their lawyers deploy same-turn self-repair operations to achieve their interactional goals based on the model of epistemic stance. More importantly, the shift of epistemic stance provides an alternative perspective to explore the choice of same-turn self-repair operations in the courtroom trial, thus enriching the growing body of literature on the study of legal discourse in Mandarin Chinese (see Ge and Wang, 2019) and same-turn self-repair operations (see Ma and Gao, 2018).

Different from the study by Szczyrbak (2021) which only centers on the use of the epistemic lexical verbs regarding certainty and doubt, the present article looks at the correlation between choice of same-turn self-repair and the shift between [K−] and [K−] in the Chinese civil courtroom interaction. In other words, judges, plaintiffs, and defendants can pick out the proper repair operations to converge or diverge their epistemic states flexibly to display their conversational actions and achieve their communicative goals.

In contrast to the research by Dong (2013) and Gibbons (2008) which examine how interactants make use of linguistic resources such as question-answer adjacency pairs in their trial narratives and debate, this article concentrates on same-turn self-repair operations and reveals that all the participants are capable of employing repair operations to demonstrate their epistemic stance in their moment-by-moment unfolding interaction.

## 5 Conclusion

### 5.1 Summary

Based on the data from 5 Chinese civil trials, this article presents a conversation analysis of the same-turn self-repair operations in Chinese civil courtroom interaction from the perspective of epistemic stance. This study attempts to build a functional and multidimensional same-turn self-repair model for judges, plaintiffs, defendants, and their lawyers in the Chinese civil courtroom based on ten operations framework (Schegloff, 2013). Altogether, six out of the ten same-turn self-repair operations appeared in the civil courtroom interaction, namely replacing (25.94%), inserting (24.84%), searching (19.6%), recycling (18.63%), deleting (8.5%), and reformatting (2.94%).

Additionally, in the interactional dynamics of Chinese civil courtroom, judges perform two basic roles, namely, controlling and moderating the talk produced by all the other participants in the courtroom and finding facts or the reality, while plaintiffs, defendants, and their lawyers offer testimony frequently in civil trials. As the co-participants of the case, plaintiffs, defendants, and their lawyers have primary epistemic access to provide repair operations. Thus, they employ more same-turn self-repairs than judges to modify their utterances to achieve communicative goals when they make claims and provide evidence in the question-answer sequences.

Besides, judges, plaintiffs, defendants, and their lawyers assert or change their epistemic stance to improve accuracy of their versions of reality, emphasize their points, or increase the credibility by deploying different operations. Judges prefer employing same-turn self-repair operations to improve the accuracy of instructions and legal interpretations, attempting to maintain the convergence [K+] epistemic stance in terms of their expertise. However, a request for information positions judges with an unknowing [K−] epistemic stance and plaintiffs, defendants, or their lawyers with a knowing [K+] epistemic stance. Therefore, when judges enquire about the trial narratives or confirm statements, they would upgrade or downgrade epistemic stance from [K+] to [K−] or [K−] to [K+] to obtain responses by deleting, reformatting, and replacing. In some cases, judges keep their [K−] epistemic stance by recycling the question to urge recipients to give responses.

Since defendants, plaintiffs, and their lawyers enjoy the [K+] epistemic stance in their narrative versions, they deploy most of same-turn self-repair operations to assert their [K+] epistemic stance to increase the credibility and accuracy of their statement, especially replacing, inserting, and searching. Besides, they choose recycling and inserting to claim their [K+] epistemic stance by highlighting their points of their narratives. On the contrary, they generally downgrade epistemic stance from [K+] to [K−] to decrease the certainty of statements to avoid judges’ questions or shirk responsibilities by inserting and reformatting to achieve epistemic adjustment.

### 5.2 Implications for theory and practice

Overall, this study explores same-turn self-repairs in Chinese civil courtroom in greater details through conversation analysis, which enriches the repair mechanism. Particularly, the features, patterns, and turn-by-turn analyses of repair operations in this study can offer valuable insights into the use of self-repairs in the Chinese institutional context. More importantly, epistemic stance is used to unveil how judges, plaintiffs, defendants, and their lawyers deploy those operations to achieve their interactional goals. The convergence or divergence epistemic stance provides an alternative perspective to explore the choice of same-turn self-repair operations in the courtroom setting.

In terms of pragmatics and discourse analysis, this study can shed light on understanding of the choice of same-turn self-repair operations to display convergence or divergence epistemic stance in the civil trial discourse. This finding suggests that co-participants can deploy same-turn self-repair to shift epistemic stance in the mundane interaction.

### 5.3 Limitations and directions for future research

Despite the contribution to the understanding of same-turn self-repairs in Chinese civil courtroom, there are several limitations of the present study. The data contain more than 50,000 words interaction in Chinese civil courtrooms, including 324 examples of self-repairs in the same turn. However, the sample data is still not big enough. In conversation analysis, the criteria to assess the sample size is not clearly defined. Hoey and Raymond (2022) state that if the conversational phenomenon such as “fourth-position repair” (Schegloff, 1992) is relatively rare, the time needed for collection increases. Another example is that the database consists of 30 videotaped conversations with aphasic Finnish speakers (Helasvuo, 2004) in one study. Obviously, five Mandarin Chinese civil trials are not adequate for both qualitative and quantitative analysis for this study, which can be considered as a limitation in this study.

On top of that, tones, rhythm, gestures, intonations, eye contact, facial expressions, and body movements, which have not been completely analyzed in the data, are equally valuable to repair initiators or responses in natural interaction for future studies. Since this study only focuses on same-turn self-repair operations, the future research can turn to examine other-initiated repair sequences in interaction. Another limitation of this study is that the research questions do not consider the fact that numbers of self-repairs vary among participants such as judges, plaintiffs, and defendants, and also differ from case to case. The comparison between participants and cases in terms of same-turn self-repairs can be one of the research orientations for our future study. A final limitation of this study is that civil courtroom interactions, procedures, or makeup might be different in Hong Kong, Macau, and Taiwan due to their own jurisdiction or legal system. For example, Hong Kong is currently a Special Administrative Region of China, and operates under the “One Country Two Systems principle. In other words, Hong Kong can retain its own legal system (Verhagena and Yamb, 2021). Consequently, the results concerning the choice of same-turn self-repair operations and the display of epistemic stance might not be applicable outside the mainland China.

## Data availability statement

The original contributions presented in the study are included in the article/supplementary material, further inquiries can be directed to the corresponding author.

## Author contributions

JX: Conceptualization, Funding acquisition, Supervision, Writing – review & editing. LG: Conceptualization, Investigation, Methodology, Writing – original draft.

## Footnotes

^1^http://tingshen.court.gov.cn/live

## Appendix A

**Simplified Transcription Symbols and Transcription Notations.**

[Left brackets indicate the starting point at which a current speaker’s talk is overlapped by another’s talk.

] Right brackets indicate the finishing point at which a current speaker’s talk is overlapped by another’s talk.

= Equal signs, one at the end of a line and one at the beginning, indicate no gap between the two lines.

(0.3) Numbers in parentheses indicate elapsed time in silence in tenths of a second.

:: Colons indicate prolongation of the immediately prior sound. The length of the row of colons indicates the length of the prolongation.

? Question marks indicate interrogative intonation.

。 Periods indicate declarative intonation.

, Commas indicate the level or sight rising intonation of incompletion in terms of syntax, prosody or pragmatics.

→ Arrows in the left-hand margin of the transcript may be used to call the reader’s attention to particular parts of the transcript. The author will inform the reader of the significance of the referent of the arrow by discussing it in the text.

## Appendix B

**Transcription notations.**

i. In the transcripts, the first line in italics is the original Mandarin utterance in Chinese characters; the second line is pinyin in Mandarin Chinese; the third line is a word-for-word gloss; and the fourth line is a vernacular English gloss.

ii. Arrows in the left-hand margin of the transcript may be used to call the reader’s attention to particular parts of the transcript. The author will inform the reader of the significance of the referent of the arrow by discussing it in the text.

## References

[ref1] Albert S. de Ruiter J. P. (2018). Repair: the interface between interaction and cognition. Top. Cogn. Sci. 10, 279–313. doi: 10.1111/tops.12339, PMID: 29749039 PMC6849777

[ref2] Bada E. (2010). Repetitions as vocalized fillers and self-repairs in English and French interlanguages. J. Pragmat. 42, 1680–1688. doi: 10.1016/j.pragma.2009.10.008

[ref3] Balcha E. B. (2014). Functional inter-textuality in the spoken and written genres of legal statutes: a discursive analysis of judge's summing-up and lawyers' closing arguments in Adama high criminal court. Stud. Logic Gramm. Rhetor. 38, 7–25. doi: 10.2478/slgr-2014-0029

[ref4] Bristol R. Rossano F. (2020). Epistemic trespassing and disagreement. J. Mem. Lang. 110:104067. doi: 10.1016/j.jml.2019.104067

[ref5] Chen M. H. Ye S. X. (2022). Extending repair in peer interaction: a conversation analytic study. Front. Psychol. 13:926842. doi: 10.3389/fpsyg.2022.926842, PMID: 36106036 PMC9466649

[ref6] Cuenca M.-J. (2023). Disagreement, epistemic stance and contrastive marking in Catalan parliamentary debate. J. Pragmat. 203, 1–13. doi: 10.1016/j.pragma.2022.11.001

[ref7] Dingemanse M. Enfield N. J. (2015). Other-initiated repair across languages: towards a typology of conversational structures. Open Linguist 1, 96–118. doi: 10.2478/opli-2014-0007

[ref8] Dong J. (2013). Interpersonal metaphor in legal discourse: modality in cross-examination. J. Lang. Teach. Res. 4, 1311–1321. doi: 10.4304/jltr.4.6.1311-1321

[ref9] Drew P. (1991). “Asymmetries of knowledge in conversational interactions” in Asymmetries in dialogue. eds. Marková I. Foppa K. (Hemel Hempstead, United Kingdom: Harvester Wheatsheaf)

[ref10] Fox B. Maschler Y. Uhmann S. (2010). A cross-linguistic study of self-repair: evidence from English, German, and Hebrew. J. Pragmat. 42, 2487–2505. doi: 10.1016/J.PRAGMA.2010.02.006

[ref11] Fox B. Wouk F. Hayashi M. Fincke S. Tao L. Sorjonen M.-L.. (2009). “A cross-linguistic investigation of the site of initiation in same-turn self-repair” in Conversation analysis: Comparative perspectives. ed. Sidnell J. (Cambridge: Cambridge University Press)

[ref12] Ge Y. Wang H. (2019). Understanding the discourse of Chinese civil trials: the perspective of critical genre analysis. J. Pragmat. 152, 1–12. doi: 10.1016/j.pragma.2019.07.024

[ref13] Gibbons J. (2008). “Questioning in common law criminal courts” in Dimensions of forensic linguistics. eds. Gibbons J. Turell M. T. (Amsterdam: John Benjamins Publishing Company)

[ref14] Grzech K. (2021). Using discourse markers to negotiate epistemic stance: a view from situated language use. J. Pragmat. 177, 208–223. doi: 10.1016/j.pragma.2021.02.003

[ref15] Han Z. (2011). The discursive construction of civil judgments in mainland China. Discourse Soc. 22, 743–765. doi: 10.1177/0957926511419924

[ref16] Hayashi M. Hayano K. (2013). “Proffering insertable elements: a study of other-initiated repair in Japanese” in Conversational repair and human understanding. eds. Hayashi M. Raymond G. Sidnell J. (Cambridge: Cambridge University Press)

[ref17] Heffer C. (2008). “The language and communication of jury instruction” in Dimensions of forensic linguistics. eds. Gibbons J. Turell M. T. (Amsterdam, John Benjamins)

[ref18] Helasvuo M.-L. (2004). Searching for words: syntactic and sequential construction of word search in conversations of Finnish speakers with aphasia. Res. Lang. Soc. Interact. 37, 1–37. doi: 10.1207/s15327973rlsi3701_1

[ref19] Heller V. (2021). Embodied displays of "doing thinking." epistemic and interactive functions of thinking displays in Children's argumentative activities. Front. Psychol. 12:636671. doi: 10.3389/fpsyg.2021.636671, PMID: 33679563 PMC7935546

[ref20] Hellermann J. (2009). Looking for evidence of language learning in practices for repair: a case study of self-initiated self-repair by an adult learner of English. Scand. J. Educ. Res. 53, 113–132. doi: 10.1080/00313830902757550

[ref21] Heritage J. (2012a). Epistemics in action: action formation and territories of knowledge. Res. Lang. Soc. Interact. 45, 1–29. doi: 10.1080/08351813.2012.646684

[ref22] Heritage J. (2012b). “Epistemics in conversation” in The handbook of conversation analysis. eds. Sidnell J. Stivers T. (Hoboken: Wiley-Blackwell)

[ref23] Heritage J. Clayman S. (2010). Talk in action: interactions, identities, and institutions. Hoboken: Wiley-Blackwell.

[ref24] Hoey E. M. Raymond C. W. (2022). “Managing conversation analysis data” in The open handbook of linguistic data management. eds. Berez-Kroeker A. L. McDonnell B. Koller E. Collister L. B. (Cambridge: The MIT Press)

[ref25] Levinson S. C. (1983). Pragmatics. Cambridge: Cambridge University Press.

[ref26] Li X. Yu H. (2021). Parataxis or hypotaxis? Choices of taxis in Chinese–English translation. Lingua 251:103026. doi: 10.1016/j.lingua.2020.103026

[ref27] Ma W. Gao Y. (2018). A study on the same-turn self-repair in Chinese doctor-patient interaction. J. Foreign Lang. 41, 42–54.

[ref28] Marin-Arrese J. (2015). Epistemicity and stance: a cross-linguistic study of epistemic stance strategies in journalistic discourse in English and Spanish. Discourse Stud. 17, 210–225. doi: 10.1177/1461445614564523

[ref29] Matoesian G. M. (1993). Reproducing rape. Cambridge: Polity Press.

[ref30] Matoesian G. M. (2018). This is not a course in trial practice: multimodal participation in objections. J. Pragmat. 129, 199–219. doi: 10.1016/j.pragma.2018.03.022

[ref31] McHoul A. W. (1990). The organization of repair in classroom talk. Lang. Soc. 19, 349–377. doi: 10.1017/S004740450001455X

[ref32] Mininni G. Scardigno R. Grattagliano I. (2014). The dialogic construction of certainty in legal contexts. Lang. Dial. 4, 112–131. doi: 10.1075/ld.4.1.07min

[ref33] Mohammad A. Al-Harahshe A. (2015). A conversation analysis of self-initiated repair structures in Jordanian spoken Arabic. Discourse Stud. 17, 397–414. doi: 10.1177/1461445615578898

[ref34] Németh Z. (2012). Recycling and replacement repairs as self-initiated same-turn self-repair strategies in Hungarian. J. Pragmat. 44, 2022–2034. doi: 10.1016/j.pragma.2012.09.015

[ref35] Ochs E. (1996). “Linguistic resources for socializing humanity” in Rethinking linguistic relativity. eds. Gumperz J. Levinson S. C. (Cambridge: Cambridge University Press)

[ref36] Pomerantz A. M. (1978). Giving a source or a basis: the practice in conversation of telling ‘how I know’. J. Pragmat. 8, 607–625. doi: 10.1016/0378-2166(84)90002-X

[ref37] Quan L. Weisser M. (2015). A study of ‘self-repair’ operations in conversation by Chinese English learners. System 49, 39–49. doi: 10.1016/j.system.2014.10.012

[ref38] Rieger C. L. (2003). Repetitions as self-repair strategies in English and German conversations. J. Pragmat. 35, 47–69. doi: 10.1016/S0378-2166(01)00060-1

[ref39] Robinson J. D. (2013). “Epistemics, action formation, and other-initiation of repair: the case of partial questioning repeats” in Conversational repair and human understanding. eds. Hayashi M. Raymond G. Sidnell J. (Cambridge: Cambridge University Press)

[ref40] Romaniuk T. Ehrlich S. (2013). “On the interactional import of self-repair in the courtroom” in Conversational repair and human understanding. eds. Hayashi M. Raymond G. Sidnell J. (Cambridge: Cambridge University Press)

[ref41] Sadock J. (2012). Formal features of questions. In Ruiter J. de (Ed.), Questions. Formal, functional and interactional perspectives Cambridge: Cambridge University Press.

[ref42] Schegloff E. A. (1992). Repair after next turn: the last structurally provided defense of intersubjectivity in conversation. Am. J. Sociol. 97, 1295–1345. doi: 10.1086/229903

[ref43] Schegloff E. A. (1993). Reflections on quantification in the study of conversation. Res. Lang. Soc. Interact. 26, 99–128. doi: 10.1207/S15327973RLSI2601_5

[ref44] Schegloff E. A. (2013). “Ten operations in self-initiated, same-turn repair” in Conversational repair and human understanding. eds. Hayashi M. Raymond G. Sidnell J. (Cambridge: Cambridge University Press)

[ref45] Schegloff E. Jefferson G. Sacks H. (1977). The preference for self-correction in the organization of repair in conversation. Language 53, 361–382. doi: 10.1353/LAN.1977.0041

[ref46] Sidnell J. (2010). Conversation analysis: an introduction. Hoboken: Blackwell-Wiley.

[ref47] Simon-Vandenbergen A.-M. Aijmer K. (2007). The semantic field of modal certainty: a Corpus-based study of English adverbs. Berlin: Mouton de Gruyter

[ref48] Stivers T. (2015). Coding social interaction: a heretical approach in conversation analysis? Res. Lang. Soc. Interact. 48, 1–19. doi: 10.1080/08351813.2015.993837

[ref49] Stivers T. Mondada L. Steensig J. (2011). “Knowledge, morality and affiliation in social interaction” in The morality of knowledge in conversation. eds. Stivers T. Mondada L. Steensig J. (Cambridge: Cambridge University Press)

[ref50] Szczyrbak M. (2021). I’m thinking and you’re saying: speaker stance and the progressive of mental verbs in courtroom interaction. Text Talk 41, 239–260. doi: 10.1515/text-2019-0145

[ref51] Verhagena M. D. Yamb J. (2021). The law of attraction: how similarity between judges and lawyers helps win cases in the Hong Kong court of final appeal. Int. Rev. Law Econ. 65:105944. doi: 10.1016/j.irle.2020.105944

[ref52] Wang Z. Fan W. Fang A. C. (2022). Lexical input in the grammatical expression of stance: a Collexeme analysis of the introductory it pattern. Front. Psychol. 12:762000. doi: 10.3389/fpsyg.2021.762000, PMID: 35069335 PMC8767053

[ref53] Wong J. Waring H. Z. (2010). Conversation analysis and second language pedagogy: a guide for ESL/EFL teachers. London: Routledge.

